# *PCDH19*-related epilepsy in mosaic males: The phenotypic implication of genotype and variant allele frequency

**DOI:** 10.3389/fneur.2022.1041509

**Published:** 2022-11-03

**Authors:** Yi Chen, Xiaoxu Yang, Jiaoyang Chen, Xiaoling Yang, Ying Yang, Aijie Liu, Xiaoli Zhang, Wenjuan Wu, Dan Sun, Zhixian Yang, Yuwu Jiang, Yuehua Zhang

**Affiliations:** ^1^Department of Pediatrics, Peking University First Hospital, Beijing, China; ^2^Center for Bioinformatics, State Key Laboratory of Protein and Plant Gene Research, School of Life Sciences, Peking University, Beijing, China; ^3^Department of Pediatric Neurology, Capital Institute of Pediatrics, Beijing, China; ^4^Department of Pediatrics, The Third Affiliated Hospital of Zheng Zhou University, Zhengzhou, China; ^5^Department of Neurology, Hebei Children's Hospital, Shijiazhuang, China; ^6^Department of Neurology, Wuhan Children's Hospital, Wuhan, China

**Keywords:** PCDH19 gene, male, mosaicism, epilepsy, phenotype, genotype, variant allele frequency

## Abstract

**Objective:**

To analyze the genotypes and phenotypes of mosaic male patients with *PCDH19*-related epilepsy (*PCDH19*-RE) and explore the correlation between genotype, variant allele frequency (VAF), and phenotypic severity.

**Methods:**

Clinical data and peripheral blood samples of 11 male mosaic patients were collected and analyzed in our study. The VAF of the *PCDH19* gene from peripheral blood was quantified using amplicon-based deep sequencing. Additional 20 mosaic male patients with *PCDH19*-RE were collected from the published literature, with 10 patients whose VAFs of the *PCDH19* gene were available for analytic purposes.

**Results:**

In our cohort of 11 patients, 10 variants were identified, and four were novel. The VAF of the *PCDH19* gene from peripheral blood ranged from 27 to 90%. The median seizure onset age was 6 months (range: 4–9 months). Clinical manifestations included cluster seizures (100%), fever sensitivity (73%), focal seizures (91%), developmental delay/intellectual disability (DD/ID, 82%), and autistic features (45%). Thirty-one mosaic male patients collected from our cohort and the literature developed seizures mostly (87%) within one year of age. Variant types included missense variants (42%), truncating variants (52%), splice variants (3%), and whole *PCDH19* deletion (3%). Among 21 patients with a definite VAF from our cohort and the literature, nine had a low VAF ( ≤ 50%) and 12 had a high VAF (> 50%). Seventy-five percent of variants from the high VAF group were missense, whereas 89% of those from the low VAF group were truncations. The median seizure onset age was 6 months in the low VAF group and 9 months in the high VAF group (*p* = 0.018). Forty-four percent (4/9) of patients from the low VAF group achieved seizure-free for ≥1 year, whereas none of the 12 patients from the high VAF group did (*p* = 0.021). DD/ID was present in 83% (10/12) of the high VAF group and 56% (5/9) of the low VAF group (*p* = 0.331).

**Conclusion:**

The predominant variant types were truncating and missense variants. Missense variants tended to have higher VAFs. Patients with a high VAF were more likely to have a more severe epileptic phenotype. Our findings shed light on the phenotypic implications of VAF in mosaic males with *PCDH19*-RE.

## Introduction

*PCDH19* (protocadherin 19) is located at Xq22.1 and contains six exons that encode six extracellular cadherins (EC) repeats with conserved calcium-binding sequences, a transmembrane domain, and a cytoplasmic domain (1). It is highly expressed in the central nervous system. Dibben et al. initially identified the *PCDH19* gene as a causative gene of epilepsy and mental retardation limited to females (EFMR) in 2008 (2). In 2009, Depienne *et al*. discovered the first mosaic deletion of the *PCDH19* gene in a male epileptic patient (3). According to their findings, *PCDH19*-related epilepsy (*PCDH19*-RE) has a unique X-linked inheritance pattern (3). Both female heterozygotes and male mosaicism were affected, but male hemizygotes were asymptomatic carriers. The hypothesis of cellular interference, which refers to the coexistence of mutant and wild-type cell populations interfering with normal cell–cell communication, was thus proposed as a key pathogenic mechanism of *PCDH19*-RE (3, 4). Hypothesis for the pathogenesis of *PCDH19*-RE also includes decreased GABA_A_ receptor function (5, 6), female-related allopregnanolone deficiency (7, 8), and blood–brain barrier dysfunction (9). Recent animal and cellular modeling shed light on the mechanism of how *PCDH19* impairment led to the disease phenotype. Mincheva-Tasheva *et al*. found a reduction in excitatory synaptic contacts and impaired network formation between *PCDH19* mutant and wild-type neurons using CRISPR-cas9 knockin and knockout mice models (10). Hoshina *et al*. further demonstrated that the mismatch between *PCDH19* and *N*-cadherin pairs affects hippocampal mossy fiber synaptic functions in mice (11). Borghi *et al*. reported that accelerated *in vitro* neurogenesis triggered a defect in the cell division plain at the neural progenitor stage in patient-derived iPSCs (12).

Data from the literature indicate that *PCDH19* is one of the six genes most frequently involved in genetic epilepsies (13). Over 400 female patients have been reported, whereas mosaic male patients are rare (14). Variant allele frequency (VAF) refers to the fraction of the number of mutant reads relative to total reads at a given locus, which is an important indicator of mosaicism. VAFs directly correlate with mutant cell fractions, and recent studies demonstrated that mosaic variants with higher VAFs tend to be spread to multiple tissues within the individual (15–17). VAF has been demonstrated to be correlated with phenotype for the carrier of mosaic variants in *SCN1A* (18, 19). However, existing scholarship in this field tends to overlook the role of VAF and stays short of establishing the correlation between VAFs and phenotypes in mosaic male patients with *PCDH19*-RE. Using data from our cohort and literature reviews, this study intends to update the genotypic and phenotypic data of mosaic males with *PCDH19*-RE and explores the correlation between genotype, variant allele frequency (VAF), and phenotypic severity.

## Materials and methods

### Participants

Eleven mosaic male patients among 133 patients with *PCDH19*-RE who attended the Pediatric Department of Peking University First Hospital from January 2013 to March 2022 were recruited for this study. Seven male patients were newly collected, and four were reported previously (20–22). Clinical information was gathered from the clinic using a genetic epilepsy registration form, including seizure onset age, seizure frequency, seizure types, fever sensitivity, developmental milestones, perinatal and family history, video electroencephalogram (EEG), brain magnetic resonance imaging (MRI), and treatment. All patients were followed up by outpatient visits or by telephone. According to the available data on full-scale developmental quotient (DQ) or intelligence quotient (IQ), the DD/ID severity was classified as normal (DQ or IQ> 85), borderline (DQ or IQ = 70-85), mild (DQ or IQ = 50-70), moderate (DQ or IQ = 30-50), and severe or profound (DQ or IQ < 30). When no DQ or IQ was available, the assessment was based on communication, daily living skills, school functioning, and adaptive behavior.

### Genetic analysis

Peripheral blood DNA was collected from the patients and their parents. *PCDH19* variants were discovered by using next-generation sequencing (NGS), which include the custom-designed epilepsy panel (*n* = 3) and whole-exome sequencing (WES, *n* = 8). The custom-designed epilepsy panel had been described in previous articles (20). For WES, the samples were sequenced on the Illumina Nova 6000 platform (Illumina, San Diego, CA, USA). Variations were verified using Sanger sequencing. The *PCDH19* isoform was referenced (NM_001184880.2, GRCh37/hg19). Sequence variants were interpreted with annotations from 1,000 Genomes, gnomAD, and ClinVar databases. Synonymous variants and single-nucleotide polymorphisms with a minor allele frequency ≥5% were removed (http://gnomad.broadinstitute.org/). For further functional annotations, CADD (https://cadd.gs.washington.edu/score) PHRED scores ≥ 15 were used and prediction scores from MutationTaster (http://www.mutationtaster.org/), PolyPhen-2 (http://genetics.bwh.harvard.edu/pph2/), and SIFT (http://sift.jcvi.org/) were incorporated to predict potential protein-damaging effects. The variants were classified and interpreted based on the American College of Medical Genetics and Genomics (ACMG) guidelines (23).

### Quantification of *PCDH19* VAFs

We used the NGS results to preliminarily estimate the VAF. We further employed amplicon-based deep sequencing (ADS) to precisely quantify the VAF of the *PCDH19* variant site (24). ADS was performed by using genomic DNA obtained from the peripheral blood of our 11 patients and their parents. The proper quantity of DNA was loaded into the flow cell, and sequencing was performed on an Illumina NextSeq. Five hundrad platform (Illumina, San Diego, CA, USA). For each detected variant, we calculated the VAF as the number of variant reads divided by the number of total reads mapped to the target position.

### Collection of cases from the literature

**Figure 1:**
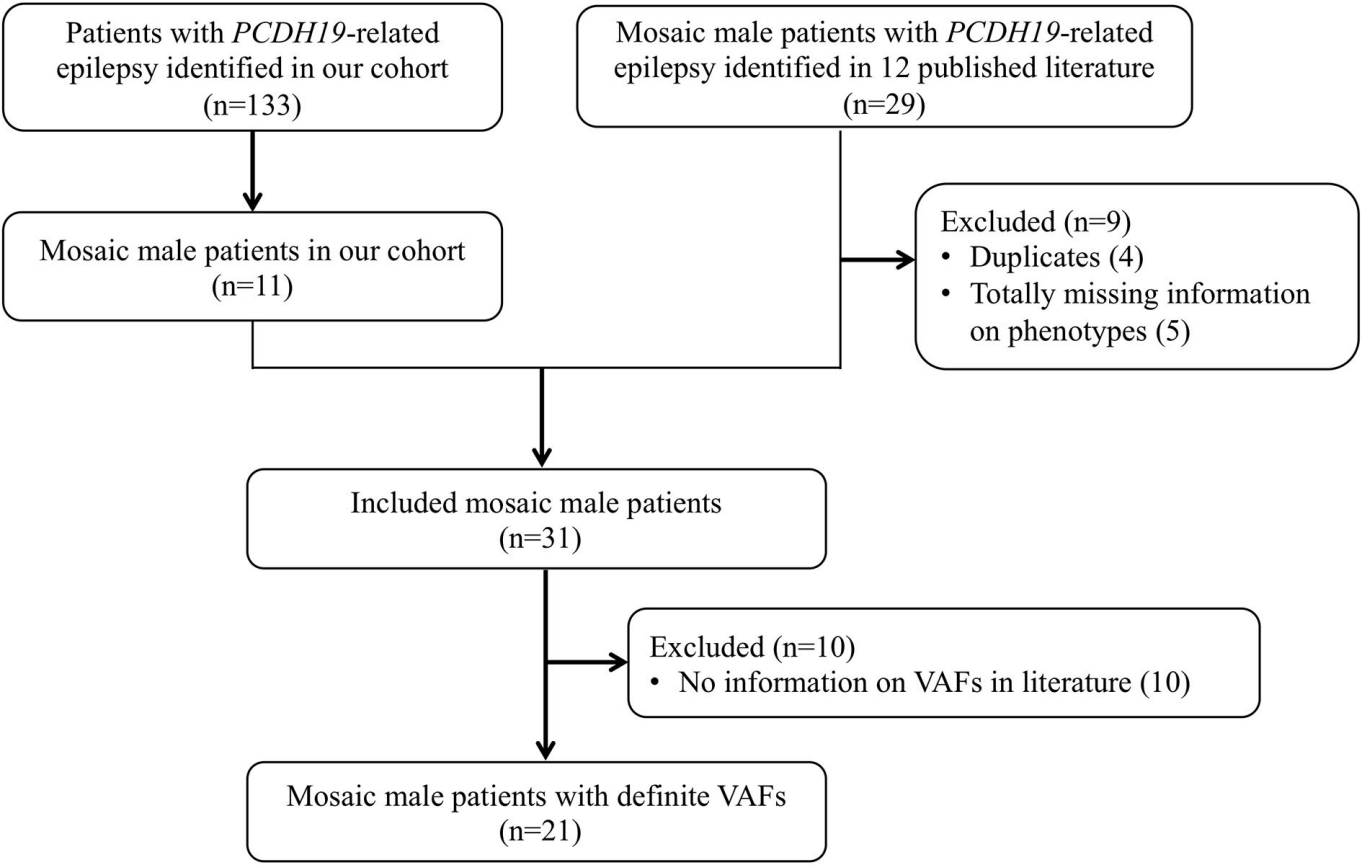
Flowchart of study sample inclusion and exclusion. A total of 31 patients were included from our cohort (*n* = 11) and the literature (*n* = 20) in the genotypic and phenotypic analysis. For further analysis of VAF and phenotype correlation, excluding not providing specific VAF in 10 patients, 21 patients were included.

We searched PubMed and Web of Science databases for relevant articles published up to August 1, 2022. All fields were searched using the terms “*PCDH19* AND epilepsy AND male AND (mosaic OR mosaicism).” Animal and cell culture studies, narrative reviews, and studies published only as conference abstracts were excluded. Twenty-nine mosaic male patients with *PCDH19*-RE were reported in 12 articles (3, 25–35), of whom four were removed owing to duplication (the same *PCDH19* variants, seizure onset age, and nationality of patients), five were removed due to completely missing phenotype data, and the remaining 20 were collected and analyzed in this study (Figure 1).

Data reported in the literature, including genetic testing methodology, *PCDH19* variant, VAF, seizure onset age, age at the last follow-up, seizure types, cluster seizures, fever sensitivity, status epilepticus, seizure frequency, DD/ID, autism spectrum disorder (ASD)/autistic feature, EEG, and treatment information, were extracted. The methods used to identify *PCDH19* variants in 14 of 20 patients were disclosed. Only 10 patients provided VAF, and VAF was calculated by targeted NGS (*n* = 5), real-time PCR (*n* = 2), WES (*n* = 1), fluorescence *in situ* hybridization (FISH, *n* = 1), and Sanger sequencing (*n* = 1). The genotypes and phenotypes of the 20 mosaic male patients with *PCDH19*-RE (Nos. 12–31) in the published literature are summarized in Supplementary Table 1.

### Statistical analysis

Phenotypic severity was evaluated by seizure onset age, seizure-free (no seizures for ≥1 year), the occurrence of DD/ID, DD/ID severity, and the occurrence of autism spectrum disorder (ASD)/autistic feature. Seizure-free was often affected by the last follow-up age, so we also compared the last follow-up age between different groups. DD/ID severity was assigned values of 0–4: (0) “normal,” (1) “borderline,” (2) “mild DD/ID,” (3) “moderate DD/ID,” and (4) “severe or profound DD/ID.” VAF of peripheral blood was classified as “low VAF” ( ≤ 50%) and “high VAF” (> 50%).

Categorical variables were expressed as percentages (%), and continuous variables were expressed as mean (standard deviations, SD) or median (interquartile range, IQR). Categorical variables were analyzed by Fisher's exact test. Continuous variables were compared by a paired samples *t*-test (if passed a *K*–*S* test) or a Wilcoxon rank-sum test.

SPSS 26.0 (SPSS Inc.) and GraphPad Prism 9.0 (GraphPad software) were used for statistical analysis. *P-*values less than 0.05 were considered statistically significant (^*^).

## Results

### Genetic and clinical profiles of our cohort patients

#### Identification of *PCDH19* variants

Ten *PCDH19* mosaic variants were identified in 11 male patients in our cohort. Variant p.Y280^*^ was found in two unrelated patients. All variants were confirmed to be *de novo*. Six were missense variants and four were truncating variants (including two nonsense and two frameshifts). Four variants, namely, p.N270K, p.E360K, p.A369P, and p.I115Kfs^*^110, were novel, of which three missense variants were classified as likely pathogenic and one truncating variant was classified as pathogenic according to ACMG guidelines. The evaluation of variant pathogenicity is shown in Table 1.

**Table 1:** Detection results and pathogenicity assessment of *PCDH19* variants in 11 male epileptic patients.

**Patient** **No**.	***PCDH19*** **variants**	**Reported** **/Novel**	**NGS**	**ADS**	**Mutation taster**	**PolyPhen-2**	**SIFT**	**CADD**	**Classification (ACMG guidelines)**
			**VAF (total** **read depth at** **variant** **position)**	**VAF (total** **read depth at** **variant** **position)**					
1	c.317T>A (p.M106K)	Reported	84.88% (86X)	80.99% (183567X)	D (1)	PD (0.923)	D (0)	D (28.4)	LP (PS2+PM2_Supporting+PP3)
2	c.1639G>C (p.A547P)	Reported	74.29% (35X)	78.10% (402842X)	D (1)	PD (0.998)	D (0)	D (24.7)	LP (PS2+PM2_Supporting+PP3)
3	c.810C>G (p.N270K)	Novel	72.48% (109X)	72.08% (143103X)	D (1)	PD (1)	D (0)	D (23.9)	LP (PS2+PM2_Supporting+PP3)
4	c.595G>C (p.E199Q)	Reported	89.19% (37X)	89.80% (167380X)	D (1)	PD (1)	D (0)	D (28.5)	LP (PS2+PM2_Supporting+PP3)
5	c.1078G>A (p.E360K)	Novel	77.50% (40X)	67.48% (47761X)	D (1)	PD (1)	D (0)	D (28.5)	LP (PS2+PM2_Supporting+PP3)
6	c.1105G>C (p.A369P)	Novel	69.66% (178X)	78.11% (68337X)	D (1)	PD (1)	D (0)	D (28.5)	LP (PS2+PM2_Supporting+PP3)
7	c.462C>G (p.Y154*)	Reported	65.38% (52X)	60.41% (43197X)	-	-	-	D (23.9)	P (PVS1+PS2+PM2_Supporting)
8	c.158dupT (p.D54Gfs*35)	Reported	32.80% (125X)	34.10% (38430X)	-	-	-	-	P (PVS1+PS2+PM2_Supporting)
9	c.344_345del (p.I115Kfs*110)	Novel	20.25% (79X)	27.46% (275210X)	-	-	-	-	P (PVS1+PS2+PM2_Supporting)
10	c.840C>A (p.Y280*)	Reported	34.27% (154X)	34.64% (336089X)	-	-	-	D (25.9)	P (PVS1+PS2+PM2_Supporting)
11	c.840C>A (p.Y280*)	Reported	31.82% (22X)	38.44% (257922X)	-	-	-	D (25.9)	P (PVS1+PS2+PM2_Supporting)

*ADS*, amplicon-based deep sequencing; *CADD*, combined annotation dependent depletion; *D*, damaging or deleterious; *NGS*, next-generation sequencing; *NA*, not available; *PD*, probably damaging; *LP*, Likely pathogenic; *P*, Pathogenic; *SIFT*, sorting intolerant from tolerant; *VAF*, variant allele frequency.

#### Quantification of *PCDH19* VAFs

The VAFs of our 11 patients were estimated to be 20–89% using NGS (total read depth range: 22–178X; mean: 82X). Furthermore, the VAFs of our 11 patients were quantified to be 27–90% using the ADS (total read depth range: 38,340–40,2842X; mean: 178,530X) (Table 1). The total read depth of ADS was significantly higher than that of NGS. However, no significant differences were observed between the two approaches in terms of VAFs (*t* = 0.487, *p* = 0.636). In summary, the median VAF was 78% (IQR: 71–83%) in six patients (Nos. 1–6) with missense variants and 35% (IQR: 31–49%) in five patients (Nos. 7–11) with truncating variants of our cohort. VAFs in our cohort of patients with missense variants were higher than those with truncating variants.

#### Clinical manifestations

The seizure onset age of 11 patients ranged from 4 to 9 months (median: 6 months). Focal seizures occurred in 10 patients (91%), generalized tonic–clonic seizures (GTCS) occurred in three patients, myoclonic seizures occurred in two patients, tonic seizures occurred in two patients, and atypical absence seizures occurred in one patient. Focal seizures in eight patients occurred mostly during sleep (Nos. 1, 2, 5, 6, 7, and 9–11). Affective symptoms were present at the start of focal seizures in four patients (Nos. 1, 5, 6, and 10). Three patients (Nos. 2, 5, and 6) had a history of convulsive status epilepticus lasting for 30 min to 1 h. All 11 patients experienced seizures in clusters. A seizure usually lasted no more than one min and was repeated more than 10 times a day and up to about 100 times a day. Most cluster episodes lasted 2–7 days. Clustering episodes mainly occurred repeatedly from 2 weeks to 3 months. Seizures in eight patients (73%) were sensitive to fever. The clinical features of 11 mosaic male patients are listed in Table 2. All patients had no family history of epilepsy.

**Table 2:** Clinical features of 11 mosaic male patients with *PCDH19*-related epilepsy in our cohort.

**Patient** **No**.	**Seizure** **onset** **age** **(mo)**	**Seizure** **types**	**Cluster** **seizures**	**Fever** **sensitivity**	**SE**	**Interictal** **VEEG**	**Age at** **the last** **follow-** **up**	**Seizure** **frequency at** **the last** **follow-up**	**Treatment**	**DD/ID**	**Autistic** **features**
1	5	Fs	Y	*N*	*N*	M	8 yrs 8 mo	1–2 months between clusters	CLB^*^,CZP,VPA,LEV, TPM,OXC,KD	Severe/profound	Y
2	6	Fs	Y	Y	Y	M	6 yrs 5 mo	1–3 months between clusters	CLB^*^,VPA,OXC,TPM, LEV,KD	Mild	Y
3	6	Fs, MS	Y	Y	*N*	M	8 yrs 6 mo	1–2 months between clusters	LTG,ZNS,CZP^*^,VPA, TPM,OXC	Severe/profound	Y
4	4	Fs	Y	Y	*N*	F	4 yrs 3 mo	1 month between clusters	CZP,VPA^*^,LTG,TPM,LEV,OXC, CBZ	Moderate	Y
5	6	Fs, GTCS, TS	Y	Y	Y	F	3 yrs 7 mo	3–4 months between clusters	**VPA,TPM,LCM,CLB**	Moderate (3yrs 7mo, DQ 39)	*N*
6	5	Fs	Y	*N*	Y	G, F	2 yrs 2 mo	1–4 months between clusters	CLB^*^,LEV^*^,VPA,LTG,TPM,NZP	Mild (1yrs 10mo, DQ 60)	*N*
7	8	Fs	Y	*N*	*N*	M	3 yrs	1–2 months between clusters	**VPA,TPM**	Moderate (3yrs, DQ 31)	*N*
8	9	GTCS, MS, AAS	Y	Y	*N*	F	14 yrs	Seizure–free for 8 yrs	VPA^*^,TPM^*^	Severe/profound (11yrs, IQ 25)	Y
9	8	Fs, GTCS, TS	Y	Y	*N*	M	2 yrs 9 mo	No seizures for 7 months	LEV,VPA^*^,OXC^*^	Moderate (2yrs 1mo, DQ 38)	*N*
10	8	Fs	Y	Y	*N*	Normal	5 yrs 4 mo	Seizure–free for 3yrs 8mo	VPA^*^,LEV^*^	Borderline (5yrs 4mo, DQ 85)	*N*
11	9	Fs	Y	Y	*N*	G, F	1yr 11 mo	No seizures for 10 months	VPA^*^,LEV^*^,OXC	Borderline (11mo, DQ 82)	*N*

*AAS*, atypical absence seizures; *CLB*, clobazam; *CZP*, clonazepam; *CBZ*, carbamazepine; *DD*, developmental delay; *Fs*, focal seizures; *F*, focal epileptic discharges; *G*, generalized epileptic discharges; *GTCS*, generalized tonic-clonic seizures; *ID*, intellectual disability; *KD*, ketogenic diet; *LCM*, lacosamide; *LTG*, lamotrigine; *LEV*, levetiracetam; *M*, multifocal epileptic discharges; *MS*, myoclonic seizures; *mo*, months; *N*, no; *NZP*, nitrazepam; *OXC*, oxcarbazepine; *SE*, status epilepticus; *TPM*, topiramate; *TS*, tonic seizures; *yr*, year; *yrs*, years; *VPA*, valproic acid; *Y*, yes; *ZNS*, zonisamide. Bold indicates the treatment used at the last follow-up.

* indicates effective treatment.

#### Neurodevelopment

All patients had a typical development before the seizure onset. Nine of 11 patients (82%) had DD/ID after seizure onset, including two mild, four moderate, and three severe/profound DD/ID. Two patients (Nos. 10 and 11) who carried the same variant p.Y280^*^ with low VAF had borderline DD/ID. Five (Nos. 1–4 and 8) of 11 patients (46%) showed autistic features.

#### Video EEG and brain MRI

Video EEG was performed on all 11 patients. Two patients (Nos. 5 and 7) had slow background activity. Interictal epileptic discharges were captured in 10 patients, including five patients (Nos. 1–3, 7, and 9) with multifocal discharges, three (Nos. 4, 5, and 8) with focal discharges, and two (Nos. 6 and 11) with focal and generalized discharges. Focal seizures were monitored in nine patients, and the site of seizure origin included the temporal (*n* = 4, Nos. 1, 5, 6, and 10), occipital (*n* = 3, Nos. 2, 5, and 11), frontal (*n* = 2, Nos. 1 and 2), central (*n* = 2, Nos. 6 and 10), parietal (*n* = 2, Nos. 7 and 11), one hemisphere (*n* = 2, Nos. 6 and 9), and anterior head (*n* = 1, No. 3) regions.

Brain MRI was normal in 10 patients. In another patient (No. 5), when he was one year old, there was a left frontal cavernous hemangioma (diameter 5.5 mm) on brain MRI.

#### Follow-up and treatment

The median follow-up time was 3 years (range: 7 months−11 years). The median age at the last follow-up was 4 years 3 months (range: 1 year 11 months−14 years). All 11 patients had tried two to seven anti-seizure medications (ASMs). Two patients (Nos. 8 and 10) achieved seizure-free for ≥1 year. Patient No. 8 at the age of 14 years has been seizure-free for 8 years and is currently on valproic acid (VPA) monotherapy. Patient No. 10 at the ages of 5 years and 4 months has been seizure-free for 3 years and 8 months and is currently on combination therapy of VPA and levetiracetam (LEV). Patient No. 9 at the ages of 2 years and 9 months had no seizures for 7 months and is currently on combination therapy of VPA, LEV, and oxcarbazepine (OXC). Patient No. 11 at the ages of 1 year and 11 months had no seizures for 10 months and is currently on combination therapy of VPA and LEV. The four patients (Nos. 8–11) mentioned above carried truncating variants with low VAF.

The remaining seven patients (Nos. 1–7) at the ages of 2 years and 2 months to 8 years and 8 months still had cluster seizures that occurred repeatedly at intervals ranging from one to four months. All seven patients (Nos. 1–7) had high VAF. Of those seven patients, six (Nos. 1–6) carried missense variants and one (No. 7) carried truncating variants.

### Genotypes and phenotypes of all 31 mosaic male patients

**Figure 2:**
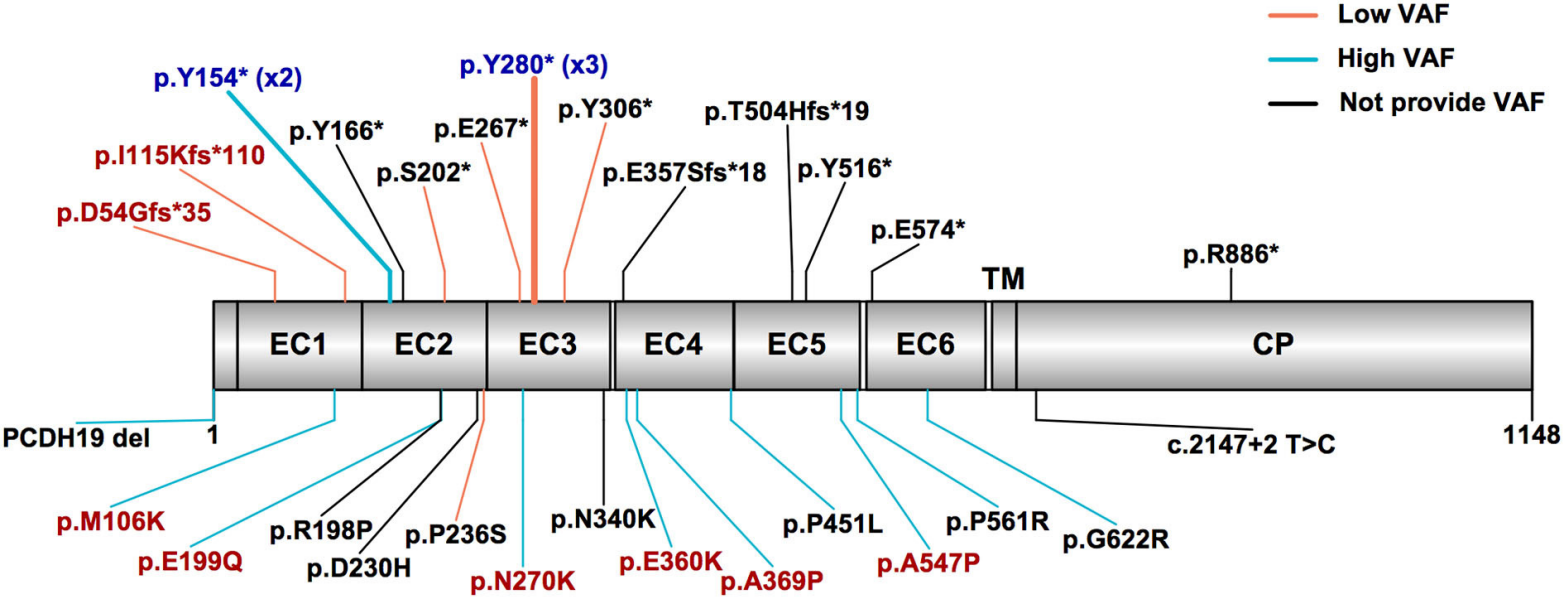
*PCDH19* variants in mosaic male patients with *PCDH19*-related epilepsy in our cohort and the published literature. The most common variant types were truncating and missense variants. Patients with high variant allele frequency (VAF > 50%) mostly carried missense variants, while patients with low VAF (VAF ≤ 50%) mostly carried truncating variants. The red text indicates variants found in our cohort patients, the black text indicates variants reported in the literature, and the blue text indicates variants reported in the literature and also found in our cohort. The truncating variants are listed above, and other variants are listed below. The orange line indicates low variant allele frequency, the sky blue line indicates high VAF, and the black line indicates not providing VAF. EC, extracellular cadherin domain, TM, transmembrane domain, CP, cytoplasmic domain.

We jointly analyzed the data collected in our cohort and reported in the literature, for a total of 31 mosaic males with *PCDH19*-RE. A schematic outlining 28 different variants in 31 mosaic male patients from our cohort and the literature is shown in Figure 2. Recurrent variants included Y280^*^ (*n* = 3) and Y154^*^ (*n* = 2). Thirteen of 31 patients (42%) carried missense variants. Sixteen patients (52%) carried truncating variants, which included 12 (75%) locating from EC1 to EC4 and four (25%) locating from EC5 to the cytoplasmic domain (Figure 2). The remaining variant was a splice variant and a whole *PCDH19* deletion. VAFs of peripheral blood in available 21 patients ranged from 10 to 100% (median: 60.2%, IQR: 34.2–78.1%).

Three male patients (Nos. 10 and 11 in our cohort and No. 18 in the literature) who carried the same variant p.Y280^*^ had low VAF (35, 38, and 22%, respectively) and presented with similar seizure onset age (8, 9, and 10 months, respectively) and mild or borderline DD/ID. Two male patients (No. 7 in our cohort and No. 19 in the literature) who carried the same variant p.Y154^*^ had high VAF (60% and 65%, respectively) and presented with a seizure onset age of 7.5 and 10 months and moderate or severe DD/ID.

The seizure onset age of *PCDH19* mosaic males ranged from 4 to 96 months (median: 8 months), of whom 84% (26/31) were within one year of age. The most prevalent seizure type was focal seizures (22/25, 88%), followed by GTCS (9/25, 36%), tonic seizures (7/25, 28%), myoclonic seizures (4/25, 16%), clonic seizures (2/25, 8%), absence seizures (1/25, 4%), atypical absence seizures (1/25, 4%), and atonic seizures (1/25, 4%). Focal seizures with affective symptoms were present in 50% of patients (10/20). Cluster seizures occurred in 100% (31/31) of patients, fever sensitivity in 75% (18/24), and history of status epilepticus in 26% (8/31). Interictal epileptic discharges were captured in 74% (17/23), including multifocal discharges (8/23), focal discharges (6/23), generalized discharges (1/23), and focal and generalized discharges (2/23). There was no significant correlation between phenotypic characteristics (including seizure type, fever sensitivity, status epilepticus, and interictal EEG pattern) and genotype (Supplementary Table 3).

The age at the last follow-up ranged from 1 year 11 months to 25 years (median: 5.5 years). At the last follow-up, 16% (5/31) were seizure-free for ≥1 year. Among 31 patients, 24 had information on ASMs therapy. The effective ASMs include VPA (9/22), LEV (8/13), clobazam (4/10), phenobarbital (3/5), clonazepam (2/5), OXC (2/12), zonisamide (ZNS, 1/2), carbamazepine (1/6), and topiramate (1/14). In four patients with seizure-free, VPA (*n* = 2), LEV (*n* = 1), and ZNS (*n* = 1) effectively controlled seizures. In another patient with seizure-free, the literature did not provide treatment information. In general, VPA and LEV were the most effective ASMs.

DD/ID occurred in 68% (21/31) of patients. The literature did not provide the DD/ID severity in three patients with DD/ID. The DD/ID severity in 28 patients ranged from normal (5/28, 18%), borderline (5/28, 18%), mild (5/28, 18%), moderate (6/28, 21%), moderate/severe (2/28, 7%), and severe/profound (5/28, 18%). ASD/autistic features were present in 55% (16/29) of patients.

### Phenotypic severity was related to VAF and variant types

First, we compared the severity of phenotypes between the patients with missense and truncating variants (Table 3). Thirteen patients carried missense variants, and 16 patients carried truncating variants from our cohort and the literature. The median seizure onset age of patients with missense variants was 6 months (IQR: 5–8 months) and of those with truncating variants was 9 months (IQR: 8–19 months). An earlier seizure onset age was observed in patients with missense variants than those with truncating variants (*p* = 0.002). The median age at the last follow-up of patients with missense variants was 5 years (IQR: 3.4–7.8 years) and of those with truncating variants was 5.7 years (IQR: 2.5–13 years; *p* = 0.809). Thirty-one percent (5/16) of patients with truncating variants were seizure-free, but none of the 13 patients with missense variants was seizure-free (*p* = 0.048). DD/ID was presented in 77% (10/13) of patients with missense variants and in 56% (9/16) of patients with truncating variants (*p* = 0.433). The median DD/ID severity was 3 (moderate DD/ID) in patients with missense variants and 1.5 (borderline to mild DD/ID) in patients with truncating variants. Patients with missense variants had a median DD/ID severity that was 1.5 units greater than patients with truncating variants, although the difference does not reach statistical significance (*p* = 0.283). ASD/autistic features were presented in 67% (8/12) of patients with missense variants and in 40% (6/15) of patients with truncating variants (*p* = 0.252).

**Table 3:** Comparison of phenotype severity expressed in mosaic male patients grouped by variant types and VAF.

**Group**	**Our cohort (*****n*** = **11) and the literature (*****n*** = **18)**			**Our cohort (*****n*** = **11) and the literature (*****n*** = **10)**		
	**Missense variants (*n* = 13)**	**Truncating variants (*n* = 16)**	***p*** **-value**	**High VAF (*n* = 12)**	**Low VAF (*n* = 9)**	***p*** **-value**
**Variant types**, ***n*****/total** ***n*** **(%)**						
Missense variants	-	-	-	9/12 (75.0%)	1/9 (11.1%)	0.003^*^
Truncating variants	-	-		2/12 (16.7%)	8/9 (88.9%)	
Seizure onset age, mo, median (IQR)	6 (5-8)	9 (8-19)	0.002^*^	6 (5–9.5)	9 (8.5–13)	0.018^*^
Age at the last follow-up, yrs, median (IQR)	5.0 (3.4–7.8)	5.7 (2.5–13.0)	0.809	6.7 (3.3–8.7)	6.0 (4.0–13.0)	0.972
Seizure-free, *n*/total *n* (%)	0/13 (0)	5/16 (31.3%)	0.048^*^	0/12 (0)	4/9 (44.4%)	0.021^*^
DD/ID, *n*/total *n* (%)	10/13 (76.9%)	9/16 (56.3%)	0.433	10/12 (83.3%)	5/9 (55.6%)	0.331
DD/ID severity 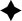 , median (IQR)	3.0 (0.5–3.8)	1.5 (1.0–3.0)	0.283	3.0 (2.0–3.5)	1.5 (1.0–2.8)	0.182
ASD/autistic features, *n*/total *n* (%)	8/12 (66.7%)	6/15 (40.0%)	0.252	7/12 (58.3%)	3/8 (37.5%)	0.650

*ASD*, autism spectrum disorder; *DD*, developmental delay; *ID*, intellectual disability; *IQR*, interquartile range; mo, months; *n*, number of patients; *VAF*, variant allele frequency; *yrs*, years. *VAF* of peripheral blood was classified as “low VAF” ( ≤ 50%) and “high VAF” (> 50%). DD/ID severity was assigned values of 0–4: (0) “normal,” (1) “borderline,” (2) “mild DD/ID,” (3) “moderate DD/ID,” and (4) “severe or profound DD/ID.” Two patients with a “moderate/severe severity of intellect in the literature were calculated as 3.5. Missing values in the literature were not included in the analysis. 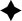
DD/ID severity: total *n* = 12 for missense variants; total *n* = 14 for truncating variants; total *n* = 12 for high VAF; total *n* = 8 for low VAF. The Wilcoxon rank-sum test or Fisher's exact test was used. Statistical significance:

* *p* < 0.05.

Second, we explored the relationship between VAF and phenotypic severity (Table 3). We classified the VAF of peripheral blood into “low VAF” ( ≤ 50%) and “high VAF” (>50%). A total of 21 patients had available VAFs from our cohort and the literature, of whom 12 had a low VAF (median: 78%, IQR: 66–88%) and 9 had a high VAF (median: 34%, IQR: 21–49%). In the high VAF group of patients, nine of 12 (75%) were detected with missense variants, two truncating variants, and one whole *PCDH19* deletion. In the low VAF group of patients, eight of nine (89%) were detected with truncating variants and one had a missense variant. Missense variants were more often seen in the high VAF group (*p* = 0.003). The earlier seizure onset age was found in the patients with high VAF compared with low VAF (median: 6 months, IQR: 5–9.5 months vs. median: 9 months, IQR: 8.5–13 months; *p* = 0.018). The median age at the last follow-up of patients with high VAF was 6.7 years (IQR: 3.3–8.7 years) and of those with low VAF was 6 years (IQR: 4–13 years; *p* = 0.972). Seizure-free occurred in 44% (4/9) of patients with low VAF, but not in all 12 patients with high VAF, indicating a significant difference between the two groups (*p* = 0.021). The percentage of DD/ID was higher in patients with high VAF than in those with low VAF (83 vs. 56%; *p* = 0.331), although statistical significance was not reached. Patients with high VAF also had a median DD/ID severity that was 1.5 units greater than patients with low VAF, though results did not reach statistical significance (*p* = 0.182). ASD/autistic features were presented in 58% (7/12) of patients with high VAF and in 38% (3/8) of patients with low VAF (*p* = 0.650).

Finally, we classified VAFs into “close to 50%” (25–75%) or “far from 50%” (< 25 or > 75%). No significant difference was observed in phenotypic severity between the two groups (Supplementary Table 2).

## Discussion

In this study, we described a cohort of 11 mosaic male patients with *PCDH19*-RE in China. The cases reported in this study added four novel variants to the *PCDH19* genotypic spectrum. This study further summarized the genotypic and phenotypic characteristics of *PCDH19-RE* in 31 mosaic male patients from our cohort and the literature. Truncating and missense variants were the most common variant types in mosaic male patients, as reported in female patients (14). Seizure onset occurred mostly during the first year of life, with a median age of 8 months in mosaic male patients. The median seizure onset age in female patients was relatively later, about 10 to 12 months (36–38). The hallmark features of *PCDH19*-RE in females were focal seizure clusters, most with affective symptoms, and often induced by fever (36, 39). These distinctive features were also observed in mosaic male patients. Overall, mosaic male patients shared similar typical clinical characteristics with female patients of *PCDH19*-RE in terms of focal seizures, seizures occurring in clusters, sensitivity to fever, DD/ID, and ASD/autistic features (13, 36, 38).

The recurrent variants p.Y280^*^ (3/31) and p.Y154^*^ (2/31) were identified in mosaic male patients. As outlined in results section, we observed that the same recurrent variant in different mosaic male patients had a similar VAF and phenotypic severity. The p.Y280^*^ variant was previously reported in a 25-year-old female patient, with seizure onset at 9 months, seizure-free at 12 years 5 months, moderate ID, and autistic features (40). The p.Y280^*^ variant seemed to have a milder phenotype in mosaic male patients. The p.Y154^*^ variant was previously reported in a 9-year-old female patient; she began having seizures at the age of 13 months and had very mild ID (41). The p.Y154^*^ variant appeared to have a relatively severe phenotype in mosaic male patients. As noted, males and females manifested a distinct phenotype severity despite having the same *PCDH19* variant site. Our observation of VAF and its phenotypic implementation in *PCDH19*-RE male patients reflected a cleaner correlation. On the contrary, X-inactivation in females could partially explain the wide variability in the phenotypic expression of PCDH19-RE in females (13, 38).

We found that mosaic male patients with missense variants had a higher VAF than those with truncating variants. The lowest VAFs of missense variants and truncating variants in the peripheral blood of male mosaic patients were observed to be 45 and 8%, respectively (31, 34). We have no clear explanation for this discrepancy. One possible explanation is that truncating variants might require a lower variant load threshold to trigger an epileptic phenotype, and further functional studies will be required to explore this phenomenon.

The main finding of our study was that mosaic male patients with missense variants were more frequently observed with high VAF, had an earlier seizure onset, and were less likely to achieve seizure-free than those with low VAF and truncating variants. Our study revealed that both variant types and VAFs significantly influenced the severity of epileptic phenotypes. To date, three cases of mosaic *PCDH19* variants in males without epilepsy have been reported (20, 32). There was a male case of mosaic *PCDH19* (missense variant E414K) with ASD, but no seizures (32). The VAF of the *PCDH19* variant was detected in peripheral blood, but not in skin fibroblasts, using Sanger sequencing, and the VAF of peripheral blood was 50%. Nonetheless, the detection limit of mosaic variants was usually approximately 10% for Sanger sequencing, by which low-frequency variants might remain undiscovered (42). We have previously reported two asymptomatic individuals with mosaic *PCDH19* (both missense variants, namely, p.V163G and p.D124N) in the fathers of girls with *PCDH19*-RE. The VAF of peripheral blood was 13 and 17%, respectively (20). Pathogenic *PCDH19* variants in the above three asymptomatic mosaic males were low VAF, but missense variants. This may mean that variant types do not play a determinant role in phenotypic severity. A previous study showed that missense variants seem to have comparable effects on epileptic phenotypes to those resulting in truncated PCDH19 protein located from EC1 to EC4 and have more severe effects on epileptic phenotypes compared with truncated PCDH19 protein located from EC5 to the cytoplasmic domain (14). Considering truncating variants were mainly located from EC1 to EC4 in mosaic male patients, we can conclude that VAFs were primarily responsible for the severity of epileptic phenotypes.

A possible explanation of our main finding is that a higher VAF leads to more severe brain dysfunction. The conclusion is based upon the assumption that VAFs in peripheral blood could be more similar to VAFs in the brain. Several recent studies have also demonstrated that mosaic variants with higher allele frequency (AF > 0.05) tend to be significantly more frequently observed in multiple tissues, including the brain (15–17) with more similar VAFs. Based on the studies presented above, we assumed that the high VAF of *PCDH19* in the peripheral blood is corresponding to the high VAF in brain tissue, which was supported by the phenotype data collected from our cohort and the literature. Our study suggests that VAF in peripheral blood is an important indicator of mosaicism and has implications for the phenotypic severity of *PCDH19*-RE.

An alternative explanation of how VAF affects phenotype is the cellular interference hypothesis, which would expect mosaic males with VAFs closer to 50% have a high level of cellular interference to express more severe phenotypes in *PCDH19*-RE (28). However, we found no evidence that phenotypes with a VAF of peripheral blood close to 50% were more severe than those with a VAF of peripheral blood far from 50%. The known cellular interference mechanisms may not wholly explain the phenotypic severity of mosaic male patients. The distribution of *PCDH19* mosaicism in the brain cells of male patients has not yet been studied due to the difficulty in obtaining brain tissue. Other mechanisms, such as decreased GABA_A_ receptor function due to the downregulation of *PCDH19*, may also play a role in the phenotypic variability in mosaic males (5, 6).

Depienne *et al*. first reported a male epileptic patient with a mosaic deletion of the whole *PCDH19* gene, and FISH calculated the VAF to be 100% in peripheral blood and 47% in skin fibroblasts (3). Terracciano *et al*. used real-time PCR to determine the VAF of two male *PCDH1*9 mosaic patients with epilepsy. The VAF of peripheral blood and urine was 90% in one patient, and the VAF of peripheral blood, oral mucosa, and hair was 10% in another (25). The VAFs in multiple tissues of our patient No. 1 were previously quantified by microdroplet digital PCR in our research group and found to be similarly high. The VAFs of peripheral blood, oral mucosa, saliva, hair, and urine were 81, 79, 81, 98, and 78%, respectively (20). We acknowledge that the sampling strategy affects the detected VAF, but as peripheral blood is a homogenized sample and clonal hematopoiesis only happens at a relatively higher age. The results of this study will not be affected given the relatively young age of recruitment (43, 44). Other tissues, in addition to peripheral blood, have been less studied in previous publications. It is difficult for us to analyze the association between the VAF of other tissues and phenotypes with limited data. This needs to be further examined in future studies.

The VAFs in reported male *PCDH19* mosaic patients were mainly estimated by the NGS method. By using ADS with an ultrahigh sequencing depth of over 10,000X in variant sites, we could make a very accurate quantification of VAF. The detection limit is roughly 0.05–0.1% for the ADS method, which can avoid missing very low VAF (24). The ADS results are more reliable, albeit there was no significant difference in the VAF measured by the ADS and the NGS in this study.

The strengths of the study included the detailed phenotype description and accurate VAF quantification in our cohort patients, a comprehensive review of mosaic male patients with *PCDH19*-RE, and a meta-analysis of the correlation between genotype, VAF, and phenotypic severity.

This study has some limitations. First, the conclusion of our study is limited by the relatively small sample size available. Further larger-scale studies are encouraged to validate our findings. Second, potential publication bias could not be ruled out.

## Conclusion

Our study extended and summarized the genotypes and phenotypes of *PCDH19*-RE in mosaic male patients. Truncating and missense variants were the most common variant types. VAF was higher in patients with missense variants than in those with truncating variants. We revealed that mosaic male patients carrying missense variants with a high VAF tended to have a more severe epileptic phenotype. Although our conclusions are based on a small cohort, our findings still warrant further studies to explore the mechanism of mosaic variants in *PCDH19*-RE.

## Data availability statement

The data presented in this study are available through Clinvar (http://www.clinvar.com/), with the following accession numbers SCV002583533 - SCV002583542. Further inquiry can be directed to the corresponding author.

## Ethics statement

The studies involving human participants were reviewed and approved by Ethics Committee of Peking University First Hospital (approval number 2012[453]). Written informed consent to participate in this study was provided by the participants' legal guardian/next of kin. Written informed consent was obtained from the individual(s), and minor(s)' legal guardian/next of kin, for the publication of any potentially identifiable images or data included in this article.

## Author contributions

YC and YZ designed this study. JC and XiaolY carried out the literature review. YY, AL, XZ, WW, DS, ZY, and YJ collected the data. YC and XiaoxY analyzed the data and prepared figures and tables. YC drafted the manuscript. XiaoxY and YZ revised the draft manuscript. YZ oversaw this study. YZ and XiaolY secured funding for this study. All authors contributed to the article and approved the submitted version.

## Funding

This study was supported by the National Natural Science Foundation for Young Scientists of China (No. 81701274) and the Key Research of the Ministry of Science and Technology of China (Nos. 2016YFC0904400 and 2016YFC0904401).

## Conflict of interest

The authors declare that the research was conducted in the absence of any commercial or financial relationships that could be construed as a potential conflict of interest.

## Publisher's note

All claims expressed in this article are solely those of the authors and do not necessarily represent those of their affiliated organizations, or those of the publisher, the editors and the reviewers. Any product that may be evaluated in this article, or claim that may be made by its manufacturer, is not guaranteed or endorsed by the publisher.
