# Supplementary material for: PCDH19-related epilepsy in mosaic males: The phenotypic implication of genotype and variant allele frequency
Source: Front Neurol. 2022 Nov 3;13:1041509. doi: 10.3389/fneur.2022.1041509 (PMC9669318; doi:10.3389/fneur.2022.1041509)
Supplement: Supplementary file 2 [file Table_2.DOCX]

**Supplementary Table 2. Comparison of phenotype severity expressed in mosaic male patients grouped by relativity to 50% VAF.**

|  | **Our cohort (n=11) and literature (n=10)** | | |
| --- | --- | --- | --- |
| **Group** | **Group 1 (n=10)** | **Group 2 (n=11)** | ***p*-value** |
| Variants types, n/total n (%) |  |  |  |
| Missense variants | 6/10 (60.0%) | 4/11 (36.4%) | 0.270 |
| Truncating variants | 3/10 (30.0%) | 7/11 (63.6%) |  |
| Age at seizure onset, mo, median (IQR) | 8.0 (5.0-10.5) | 8.0 (6.0-9.0) | 0.915 |
| Age at last follow-up, yrs, median (IQR) | 6.7 (4.0-9.8) | 5.5 (3.0-10.0) | 0.622 |
| Seizure-free, n/total n (%) | 1/10 (10.0%) | 3/11 (27.3%) | 0.586 |
| DD/ID, n/total n (%) | 7/10 (70.0%) | 8/11 (72.7%) | 1.000 |
| DD/ID severity^🟆^, median (IQR) | 2.0 (1.5-3.1) | 3.0 (1.0-3.6) | 0.248 |
| ASD/autistic features, n/total n (%) | 6/10 (60.0%) | 4/10 (40.0%) | 0.656 |

Group 1 refers to the patients with VAF as “far from 50%” (<25% or >75%); Group 2 refers to the patients with VAF as “close to 50%” (25%-75%). ASD, autism spectrum disorder; DD, developmental delay; ID, intellectual disability; IQR, interquartile range; mo, months; n, number of patients; VAF, variant allele frequency; yrs, years. DD/ID severity was assigned values of 0-4: (0) “normal”, (1) “borderline”, (2) “mild DD/ID”, (3) “moderate DD/ID”, and (4) “severe or profound DD/ID”. Two patients with a “moderate/severe” level of intellect in the literature were calculated as 3.5. Missing values in the literature were not included in the analysis. ^🟆^DD/ID severity: Total n=10 for group 1; Total n=10 for group 2. The Wilcoxon rank-sum test or Fisher’s exact test was used. Statistical significance: **p* < 0.05.
