# Supplementary material for: PCDH19-related epilepsy in mosaic males: The phenotypic implication of genotype and variant allele frequency
Source: Front Neurol. 2022 Nov 3;13:1041509. doi: 10.3389/fneur.2022.1041509 (PMC9669318; doi:10.3389/fneur.2022.1041509)
Supplement: Supplementary file 3 [file Table_3.DOCX]

**Supplementary Table 3. Comparison of phenotypic characteristics expressed in mosaic male patients grouped by variant types and VAF.**

|  | **Our cohort (n=11) and the literature (n=18)** | | |  | **Our cohort (n=11) and the literature (n=10)** | | |
| --- | --- | --- | --- | --- | --- | --- | --- |
| **Group** | **Missense variants (n=13)** | **Truncating variants**  **(n=16)** | ***p*-value** |  | **High VAF**  **(n=12)** | **Low VAF**  **(n=9)** | ***p*-value** |
| Focal seizures, n/total n (%) | 10/10 (100%) | 10/13 (76.9%) | 0.229 |  | 12/12 (100%) | 7/9 (77.8%) | 0.171 |
| Generalized tonic-clonic seizures, n/total n (%) | 3/10 (30.0%) | 4/13 (30.8%) | 1.000 |  | 3/12 (25.0%) | 4/9 (44.4%) | 0.397 |
| Tonic seizures, n/total n (%) | 3/10 (30.0%) | 4/13 (30.8%) | 1.000 |  | 3/12 (25.0%) | 2/9 (22.2%) | 1.000 |
| Myoclonic seizures, n/total n (%) | 1/10 (10.0%) | 1/13 (7.7%) | 1.000 |  | 2/12 (16.7%) | 1/9 (11.1%) | 1.000 |
| Status epilepticus, n/total n (%) | 4/13 (30.8%) | 2/16 (12.5%) | 0.364 |  | 5/12 (41.7%) | 2/9 (22.2%) | 0.642 |
| Fever sensitivity, n/total n (%) | 8/10 (80.0%) | 8/12 (66.7%) | 0.646 |  | 9/12 (75.0%) | 8/8 (100%) | 0.242 |
| Interictal epileptic discharges of EEG,  n/total n (%) | |  |  |  |  |  |  |
| Multifocal discharges | 4/10 (40.0%) | 4/12 (33.3%) | 1.000 |  | 5/11 (45.5%) | 2/9 (22.2%) | 0.374 |
| Focal discharges | 4/10 (40.0%) | 4/12 (33.3%) | 1.000 |  | 4/11 (36.4%) | 3/9 (33.3%) | 1.000 |
| Generalized discharges | 1/10 (10.0%) | 2/12 (16.7%) | 1.000 |  | 1/11 (9.1%) | 2/9 (22.2%) | 0.566 |

n, number of patients; VAF, variant allele frequency. VAF of peripheral blood was classified as “low VAF” (≤50%) and “high VAF” (>50%). Missing values in the literature were not included in the analysis. Fisher’s exact test was used. Statistical significance: ^*^*p* < 0.05.
